# Lessons for Emergency Surgery in the Second Wave: One-Month Single-Centre Experience During the First Wave of COVID-19

**DOI:** 10.7759/cureus.12685

**Published:** 2021-01-13

**Authors:** Taner Shakir, Muhammad Rafaih Iqbal, Nourelhuda M Darwish, Naveed Kirmani

**Affiliations:** 1 General Surgery, Princess Alexandra Hospital NHS Trust, Harlow, GBR

**Keywords:** covid-19, emergency surgery

## Abstract

Introduction

The global COVID-19 pandemic had a deleterious effect upon elective and emergency surgery. Focus of patient care was directed to emergency services. Association of Surgeons of Great Britain and Northern Ireland guidelines advised a trend towards conservative management. Traditional surgical intervention was reserved only for selected cases only. We evaluated our emergency practice over a four-week period during the first peak of COVID-19.

Methods

A retrospective single-centre analysis was performed of consecutive patients seen by the emergency general and vascular surgery on-call team in a District General Hospital over a four-week period (30 March 2020-26 April 2020). Primary outcome was 30-day COVID-19 mortality. Secondary outcomes were 30-day complications, readmission rate and non-COVID-19-related mortality. Adherence to intercollegiate guidelines was also assessed.

Results

A total of 184 patients were assessed during the period. The median age was 55 years (interquartile range 34-75), with a male:female ratio of 1:0.7. Thirty-day COVID-19- and non-COVID-19-related mortalities were 3% and 8%, respectively. Thirteen percent of patients developed complications and 9% represented to the emergency department within 30 days. Conservative management was initially employed in 78% of patients. This had success rates in appendicitis and cholecystitis of 72% and 75%, respectively. A CT thorax was included in 89% having a CT abdomen and pelvis. Thirty-eight percent had a COVID-19 polymerase chain reaction (PCR) swab test performed throughout the study period. Fifty-two percent of individuals who underwent emergency surgery had a swab performed prior to operative intervention.

Conclusions

Conservative management seems to be reasonably effective and may re-shape the way we treat a proportion of surgical pathologies in the future. Further long-term data are required in order to evaluate this. A paucity of PCR testing was due to nationwide capacity shortcomings. This must be addressed in future peaks with rapid testing in order to triage patients to the appropriate setting.

## Introduction

The global COVID-19 pandemic, since its origin in Wuhan [1], had a deleterious effect upon elective and emergency surgery. The first peak of the virus caused a significant disruption in planned care, with redistribution of operating theatres for utilisation as intensive care beds [2]. The entire focus of patient care shifted from elective to emergency [3]. About 28 million elective operations globally were cancelled during the peak 12 weeks of the pandemic, with approximately 2.3 million cancellations per week [4].

As the focus shifted on to emergency care of patients, there was a decline in the emergency operating as well, with a heightened focus on conservative management. The Association of Surgeons of Great Britain and Northern Ireland (ASGBI) published guidelines [5] with regard to delivering emergency General Surgery service during the COVID-19 pandemic. The emphasis was on increasing ambulatory services in order to reduce the hospital attendances and bed occupancy, in addition to attempting conservative management of non-life-threatening conditions like appendicitis and cholecystitis. Traditional surgical intervention was reserved only for extreme cases where conservative management was not possible. Hospital attendances were vastly reduced for non-COVID-related illnesses. In order to preserve capacity and avoid unnecessary inpatient exposure, more patients were managed through ambulatory services [6]. 

A lockdown in the United Kingdom during the first wave of COVID-19 was imposed on 23 March 2020 following a rapid surge in cases [7]. Our District General Hospital (DGH) halted all elective activities on this date. We evaluated our emergency practice over a four-week period during the first peak of COVID-19 in order to learn lessons for the second wave.

## Materials and methods

Study design

This was a retrospective review of prospectively maintained database of all the consecutive emergency patients referred to the on-call General and Vascular Surgery team.

Setting

Our DGH offers services to a local population of about 500,000 residents in West Essex and East Hertfordshire. It was one of the worst-hit areas with COVID-19 during the first wave. 

Time Period

Four weeks (30 March 2020-26 April 2020)

Outcomes

Primary Outcome

The primary outcome was 30-day COVID-19-related mortality.

Secondary Outcomes

The secondary outcome was 30-day complications, readmissions and non-COVID-19-related mortality.

Data collection

Data were collected from hospital electronic records and patient notes. Data collected included patient demographics, presenting symptoms, management (conservative vs operative), COVID-19 polymerase chain reaction (PCR) swab results, CT Thorax, 30-day complications, readmissions and mortality. Complications were classified as per the Clavien-Dindo classification [8]. Both COVID-19- and non-COVID-19-related mortality data were collected. All the data were independently checked and verified by two authors.

Pathway

All the patients presenting to the Accident and Emergency department were triaged for referral to the appropriate specialty. The patients were seen by the Surgical Registrar/Surgical Consultant using the standard personal protective equipment (PPE) as per the guidelines. The initial UK PPE guidelines were published on 2 April 2020 [9], which recommended the use of gowns instead of aprons, mandatory eye protection and guidance on the use of FFP3 masks. Additional updates on PPE were released on 10 April 2020 [10]. Appropriate decisions were made with regard to the further management of the patient regarding ambulatory care, admission and surgery. 

Patients requiring computed tomography (CT) scan of the abdomen and pelvis also had a CT thorax to assess for COVID-19. COVID-19 PCR swabs were not conducted routinely on patients presenting to the hospital until towards the end of the study period. Furthermore, once routine swabbing was mandatory, limited testing capacity translated to results, taking up to 72 hours to process. The majority of swabs were outsourced; however, two slots were available for in-house processing. This had a six-hour turnaround time and was helpful in trying to plan urgent surgery.

Standard PPE precautions were taken intra-operatively. Laparoscopy was avoided due to uncertainties regarding aerosolisation of peritoneal contents such as blood and peritoneal fluid. 

Ethical considerations

Local hospital ethics committee exempted this study from ethical approval and was registered as an audit.

## Results

**Figure 1:**
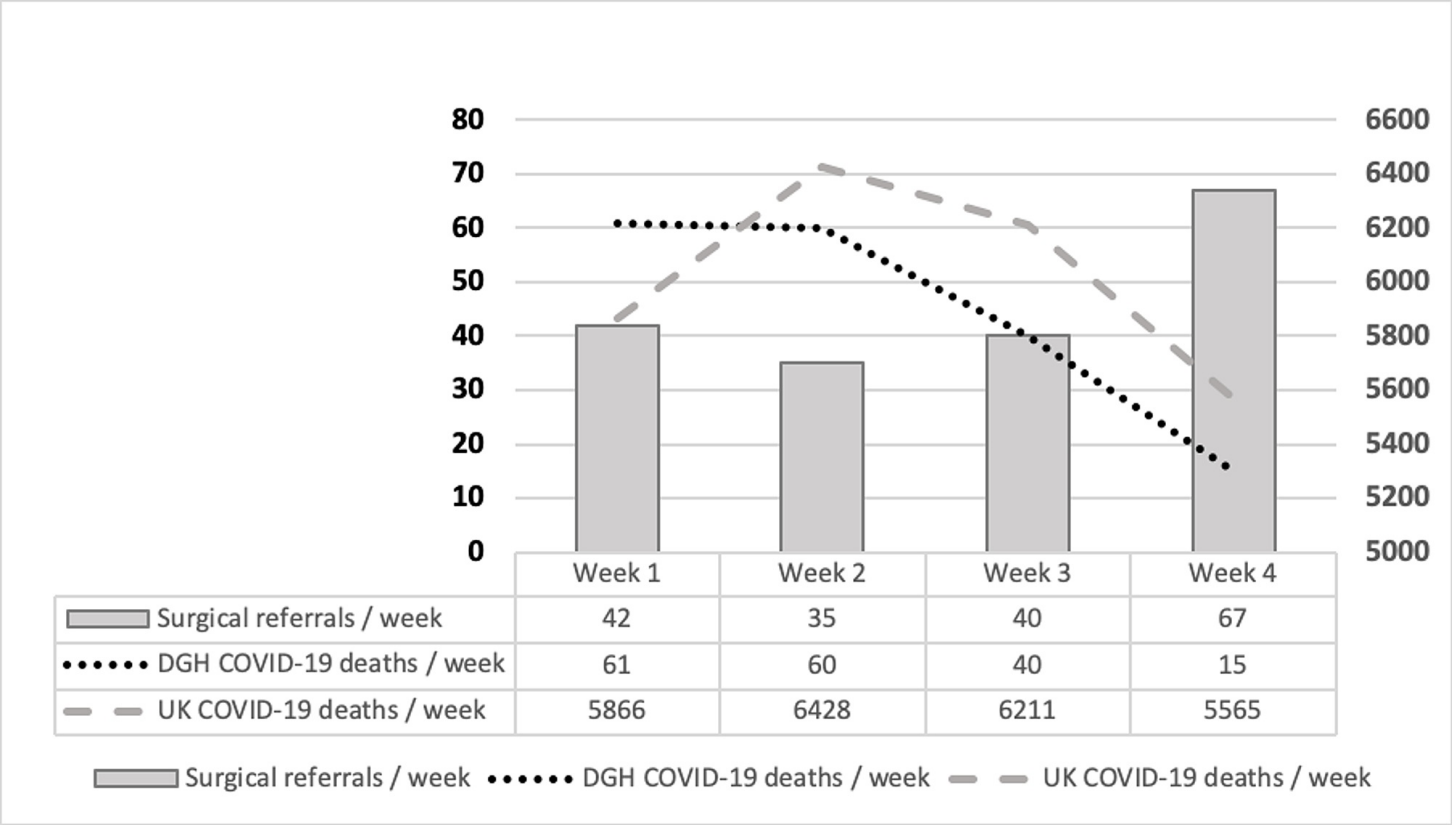
Comparison of Surgical Referrals With DGH and UK Deaths per Week. District General Hospital (DGH)

A total of 184 patients were assessed during the study period. Ninety-three percent (171/184) were General Surgical, whereas 7% (13/184) were Vascular Surgery. Weekly referrals were constant until week 4, where there was a 68% increase from week 3 (Figure 1). Median age was 55 years (interquartile range (IQR) 34-75 years). Male-to-female ratio was 1:0.7.

Sixteen (9%) patients represented to the Emergency department within 30 days of index discharge (Table 1). Twenty-three (13%) patients developed complications within 30 days (Table 2). Thirty-day COVID-19- and non-COVID-19-related mortalities were observed in 5 (3%) and 14 (8%) patients, respectively. Of the COVID-19-related mortalities, 100% were male with a mean age of 79. The original presentations were perforated appendicitis, abdominal aortic aneurysm (AAA) rupture, perforated duodenal ulcer, pancreatitis and dry gangrene of a lower limb.

**Table 1:** Causes of Representation Within 30 days Acute Kidney Injury (AKI), Small Bowel Obstruction (SBO), Endoscopic Retrograde Cholangiopancreatography (ERCP)

Representation within 30 days (n = 16, 8.7%)	
Original presentation	Representation cause
Abdominal mass	Hyponatremia
Abdominal pain	Worsening pain
Appendicitis	Worsening pain
Appendicitis	Worsening pain
Cholecystitis	Worsening pain
Cholecystitis	Ascites
Cholecystitis	Worsening pain
Cholecystitis	Cholecystitis
Gallstone pancreatitis	Elective ERCP
Obstructive uropathy	AKI
Pancreatic collection	Necrotizing pancreatitis
Pancreatitis	Worsening pain
Portacath displaced	AKI, SBO
Recurrent sigmoid volvulus	Myocardial infarction
Sigmoid volvulus	Recurrent sigmoid volvulus
Subdural haematoma	AKI

**Table 2:** Thirty-Day Clavien-Dindo Complications and Grade

Clavien-Dindo Grade	Cause	N , %
I	Worsening pain	1, 0.5
	Wound dehiscence	1, 0.5
	Surgical-site infection	1, 0.5
	Pressure ulcer	1, 0.5
II	Pneumonia	3, 1.6
	AKI	6, 3.3
IIIa	Post-operative collection	2, 1.1
	Recurrent sigmoid volvulus	1, 0.5
IIIb	Appendicitis failed conservative management	1, 0.5
IVa	N/A	0, 0
IVb	Necrotizing pancreatitis	1, 0.5
V	COVID-19 pneumonitis	5, 2.7
	Non-COVID-19 mortality	14, 7.6

**Figure 2:**
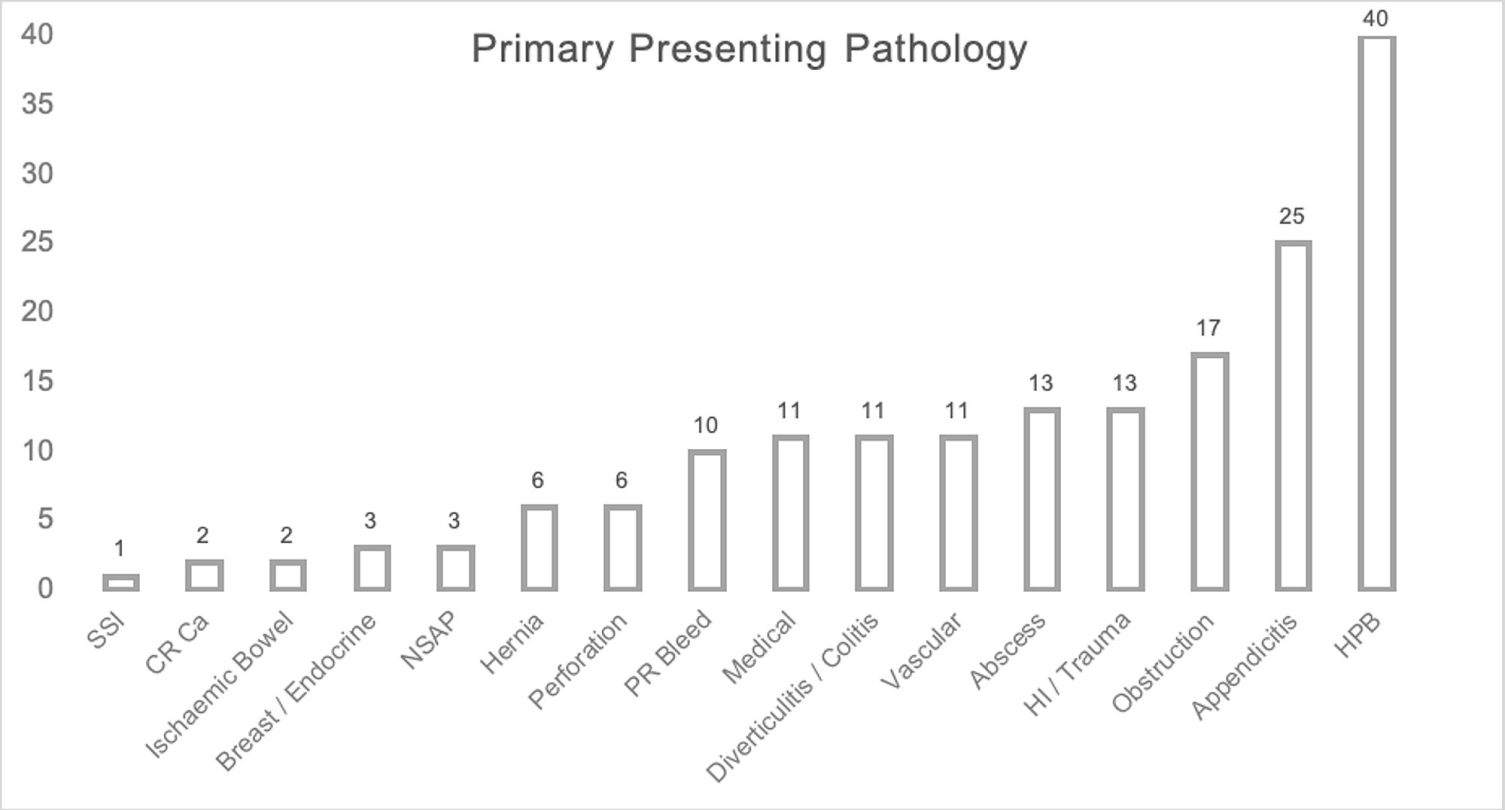
Primary Presenting Pathology. SSI (Surgical Site Infection), CR Ca (Colorectal Carcinoma), NSAP (Non-Specific Abdominal Pain), PR Bleed (Per Rectal Bleeding),  HI (Head Injury), HPB (Hepatobiliary – to Include Biliary Colic, Cholecystitis, Pancreatitis)

**Figure 3:**
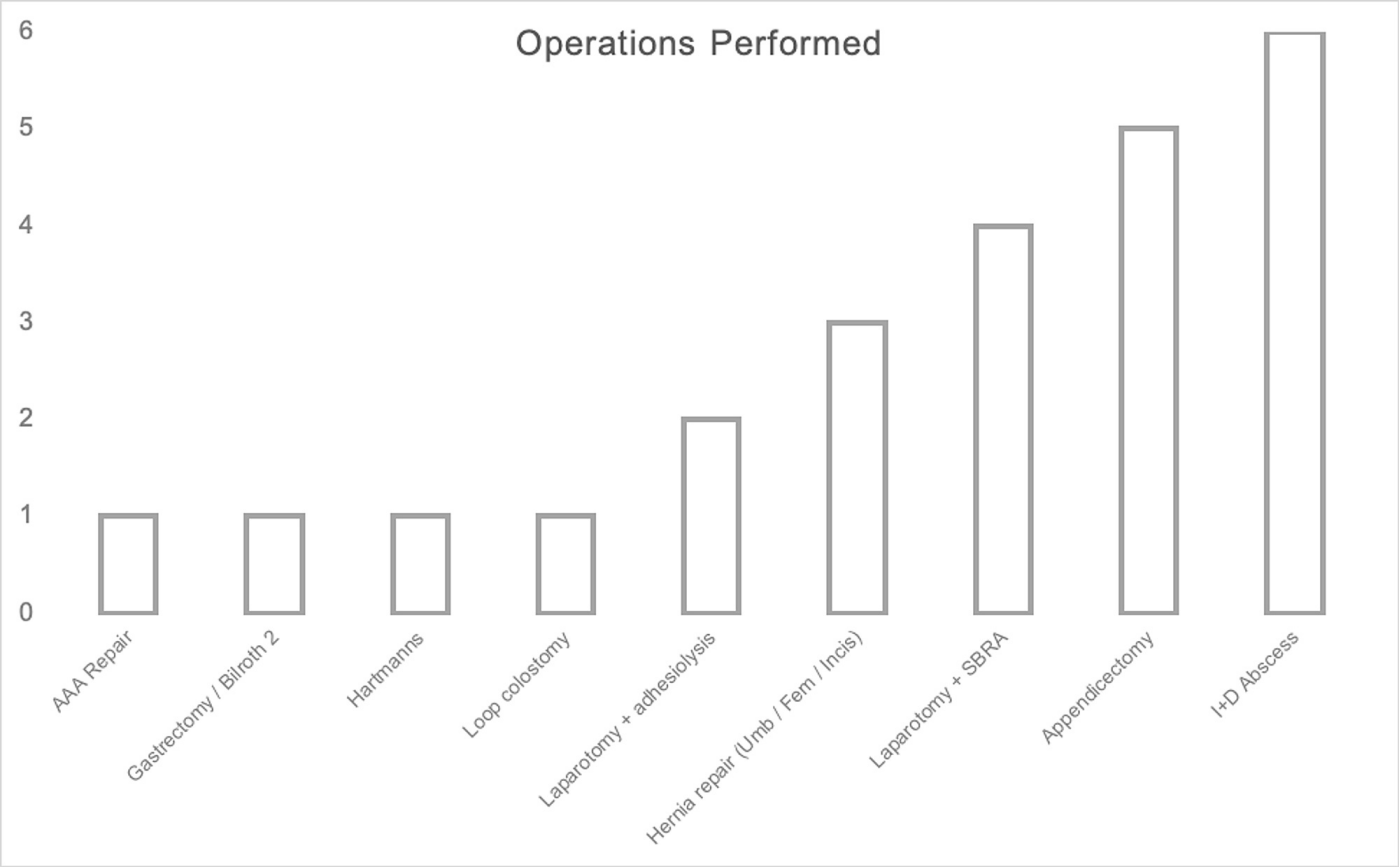
Emergency Operations Performed. AAA (Abdominal Aortic Aneurysm), Hernia Repair – Including Umbilical, Femoral and Incisional, SBRA (Small Bowel Resection + Anastomosis), I+D (Incision and Drainage)

The primary pathologies were hepatobiliary (n = 45, 24%), appendicitis (n = 25, 14%) and bowel obstruction (n = 17, 9%) (Figure 2). Conservative management was initially attempted in 78% of patients. Fourteen percent had initial surgical management. The main operations performed were incision and drainage (I+D) of abscess, appendicectomy and laparotomy with small bowel resection (Figure 3). Radiological and endoscopy procedures were employed in 3% of patients, respectively, in each. Interventional radiology drained three patients with collections and performed angioplasties of two ischaemic limbs. Endoscopy was utilised in the management of three patients with obstructive jaundice and two patients with rectal bleeding. Three (2%) patients were transferred to tertiary specialist centres: one arterial injury, one trauma and one peripancreatic collection. 

A total of 94 patients had a CT scan of abdomen and pelvis. Eighty-four (89%) of these patients had a CT thorax included as workup for evaluation of pneumonitis. 

Overall, a COVID-19 PCR swab was performed in 38% (70/184) of the patients presenting to the surgical team during the study period. Eight percent (14/184) of patients tested positive. Three of these patients had operations: formation of loop colostomy, AAA repair, and I+D abscess. Fifty-two percent (13/25) of the patients undergoing emergency surgery were swabbed in the pre-operative period. Twenty percent (5/25) had a negative PCR swab, while in 32% (8/25) the swab result was not reported prior to surgery. 

Cholecystitis

Sixteen patients presented with biliary colic and/or cholecystitis. Their median age was 56 years (IQR 37-77 years). Conservative management was attempted in all patients initially. Four patients were readmitted: two of them were again conservatively managed for cholecystitis; the other two needed endoscopic retrograde cholangiopancreatography for obstructive jaundice. No patient was readmitted with pancreatitis. Overall, 75% of biliary patients were successfully managed conservatively.

Appendicitis

Eighteen patients presented with diagnosed appendicitis. Their median age was 20 years (IQR 14-32). One patient was operated upon in the first instance. The remaining 17 patients were attempted to be managed conservatively. Four of these (22%) failed conservative management and required open appendicectomy. Overall, 72% of patients were successfully managed conservatively.

## Discussion

The number of people presenting to the hospital with non-COVID-19-related problems during the first peak of COVID-19 was dramatically reduced. It seemed that younger patients were more likely to present, possibly due to the older population shielding as they were more co-morbid, and due to the negative stigma around hospitals at the time. Over the data collection period, there were 1,533 patients tested for COVID-19 in our hospital, with 496 of these testing positive and 180 deaths. 

Pathologies were variable; however, the most common were hepatobiliary issues including biliary colic, cholecystitis and pancreatitis. Appendicitis, bowel obstruction, trauma and abscess were the next most common presentations. Two colorectal cancers presented during the period. A factor contributing to this may have been avoidance of seeking medical attention for potential symptoms. However, decreased endoscopy capacity should have resulted in fewer new diagnoses of cancer. One would then expect a greater number of obstructing lesions to present in the emergency setting. Again, this was not seen and may have been secondary to patient avoidance of the hospital setting. 

Operative intervention was reserved for unwell patients, or individuals not responding to conservative measures. Incision and drainage of abscesses were the most common procedure. Their presentation to secondary care is often preceded by failure of antibiotics in primary care. Thus, conservative measures are rarely effective, explaining why this was the mode of operation. Moreover, appendicitis was attempted to be managed conservatively in the first instance in all but one patient. Four patients returned, having failed antibiotic therapy, and needed open appendicectomy. 

Laparotomies were performed mainly in patients with bowel obstruction where conservative management failed or where emergent surgery was necessary. Small bowel resections were performed for bowel ischaemia, which may have been associated with less morbidity if conservative measures were not attempted initially. One Hartmann’s procedure was performed for perforated diverticular disease with four-quadrant faeculent peritonitis. This not only highlights that patients were presenting later, but also that there was only one operation for diverticulitis, suggesting a good response to conservative measures. ASGBI guidelines were followed in obstructing cancers, with a loop colostomy performed for obstructing sigmoid cancer. The patient was then managed through a multidisciplinary team approach as an outpatient. Pre-operatively, the patient was moved to a suspected COVID ward, due to dyspnoea secondary to abdominal distension, which further highlights the need for rapid swab results in the Emergency department. 

The vast majority of patients had a screening CT thorax when abdominal surgery was planned. A total of 10 scans were performed where the chest was not added. One factor responsible for this was that the Emergency department would often request imaging directly without knowing the current intercollegiate guidelines. Moreover, soon after the guidelines were announced, in the initial period of the study, there may have been poorer adherence due to lack of common knowledge amongst the surgical team. Only one patient was found to have COVID-related changes found incidentally using this screening. This questions the diagnostic value in relation to the extra radiation dose.

Almost one-third of patients did not have a COVID-19 PCR swab result before having an operation as they were not done routinely on asymptomatic patients until later in the study period.

Cholecystitis and appendicitis, encompassing the two most prominent pathologies, showed good response rates to conservative management. During the 30-day follow-up period, there were no patients returning with pancreatitis after an episode of cholecystitis. All patients were able to be managed non-operatively, with no emergency cholecystectomies required. Appendicitis was also able to be managed conservatively in the first instance in the majority of patients. This mirrors the interim studies performed to date [11]. 

In a recent study [12], a 30-day mortality of 23.8% was reported with increased mortality associated with emergency surgery as compared to elective surgery (odds ratio 1.67, 95% CI 1.06-2.63, p = 0.026). Overall 30-day mortality in our study was 11%. Complications were mainly Clavien-Dindo grade II, including pneumonia and acute kidney injury requiring antibiotics and intravenous fluid therapy, respectively. Five patients developed COVID-19 pneumonitis, with resultant mortality. 

Learning from the first wave

Emergency surgery during a global pandemic is fraught with potential problems. Operating on positive cases can be extremely time-consuming and potentially harmful due to aerosolisation risks. Theatre set-up is more complex and often requires the transfer or equipment or staff to a designated dirty theatre. Minimal personnel are inside the operating room, and when combined with wearing full PPE and air flow systems, means communication and efficiency are hindered. Turnover time after the case is also prolonged, with deep cleans and wait times necessary. Overall, operative intervention should be avoided where possible.

It is apparent that conservative management can be an effective treatment modality. This is at least in the short term but requires extended follow-up data to determine whether this translates to any long-term outcome difference.

Technologies for testing have advanced since the first wave. Emergency departments have access to rapid testing with results available in 90 minutes [13]. These should be utilised for all patients with a suspected acute abdomen. This can then aid triage to clean wards and theatres. In addition, this should negate the necessity of adding CT thorax when scanning a patient's abdomen. Furthermore, the diagnostic yield of this was shown to be negligible in a multicentre retrospective cohort study [14]. 

## Conclusions

Overall, care should be taken when considering emergency surgery during the COVID-19 pandemic. Conservative measures should be employed in the first instance where possible. Increased use of ambulatory services can help ease the burden of inpatient beds. Preventative equipment must be used according to local policy. Rapid testing should be utilised for potential surgical patients. Further follow-up data are needed for patients managed conservatively, as this may become commonplace in years to come. 
